# Molecular identification and genetic diversity analysis of *Cryptosporidium* spp. infecting dogs from central and northern Jordan: Detection of zoonotic genotype IId

**DOI:** 10.1371/journal.pone.0314462

**Published:** 2025-02-06

**Authors:** Rami M. Mukbel, Eman M. Etoom, Haifa B. Hammad, Heidi L. Enemark, Marwan M. Abu Halaweh

**Affiliations:** 1 Department of Basic Medical Veterinary Sciences, Jordan University of Science & Technology, Irbid, Jordan; 2 Department of Animal and Veterinary Sciences, Aarhus University, Tjele, Denmark; 3 Department of Biotechnology & Genetic Engineering, Philadelphia University, Amman, Jordan; Academic Medical Center: Amsterdam UMC Locatie AMC, NETHERLANDS, KINGDOM OF THE

## Abstract

*Cryptosporidium* spp. are common causes of gastrointestinal disease in both humans and animals. This was a cross-sectional study conducted to determine the infection rate and genetic characteristics of *Cryptosporidium* infecting dogs in Jordan. A total of 249 faecal samples were collected from stray, pet, and breeding dogs from kennels (independent of their clinical condition) across three governorates in Jordan (Amman and Zarqa in Central Jordan and Irbid in Northern Jordan). Faecal samples were screened for *Cryptosporidium* using polymerase chain reaction (PCR) targeting the *18S rRNA* gene, revealing an overall infection rate of 18.9% (47 out of 249). Cryptosporidiosis was significantly associated with indoor dogs, dogs cohabiting with other animals, and consuming raw food. Among the successfully sequenced samples, 25 (58.1%) were *Cryptosporidium canis*, 15 (34.9%) were *Cryptosporidium parvum*, and three (7.0%) were *Cryptosporidium baileyi*. Multiple diversity tests were employed, indicating low genetic differentiation between the studied populations of *C*. *parvum* and *C*. *canis*. Stability was observed for *C*. *parvum*, with minimal expansion observed for *C*. *canis*. Notably, each species exhibited a single dominant haplotype, consistent with the AMOVA results, where most of the variability occurred within populations. Further genotyping of *C*. *parvum* and *C*. *canis* was conducted by sequencing the *gp60* gene. *C*. *parvum* isolates worldwide displayed solely the zoonotic IId genotypes, namely, IIdA20G1, IIdA22G1, IIdA18G1, and IIdA19G1. In contrast, the *C*. *canis* isolates exhibited the animal subtypes XXe and XXd. Consequently, dogs may serve as a source of infection with *C*. *parvum* and pose a public health risk in Jordan.

## Introduction

*Cryptosporidium* spp. are considered major etiological agents of diarrhoea in humans and animals, particularly in young and immunocompromised individuals. Between 2011 and 2016, these parasites were responsible for 63% of waterborne outbreaks of protozoan diseases worldwide [1]. Yearly, *Cryptosporidium* spp. have been reported to cause eight million cases of foodborne infection [2]. Companion animals can act as asymptomatic carriers of this protozoan parasite [3]. In a study conducted in Italy, 86.8% of public parks were found to be contaminated with dog feces containing *Giardia* and *Cryptosporidium* [4], which suggests the important role of dogs in environmental contamination [5].

*Cryptosporidium* species identification included oocyst morphology, host specificity, genetic classification, and compliance with the International Code of Zoological Nomenclature (ICZN) [6]. The *Cryptosporidium* oocyst lacks distinctive morphological features to differentiate species. Therefore, molecular approaches, including conventional, nested and qPCR techniques, are used for isolate identification and biological characterisation of *Cryptosporidium* [7–9]. Depending on sequence polymorphisms, variable sites in the small subunit ribosomal RNA, acetyl-CoA synthetase, heat shock protein 70, 60-kilodalton glycoprotein, and the *Cryptosporidium* oocyst wall protein genes can be targeted to discriminate between species and subspecies [6, 10, 11].

Among the species causing infection in humans, *Cryptosporidium hominis* and *Cryptosporidium parvum* are the most common. The latter is known to be transmitted from animals to humans, particularly from calves serving as the primary reservoir. In contrast, *C*. *hominis* is considered an anthroponotic species [12].

Dogs are most commonly infected with *Cryptosporidium canis*, although they can also contract *C*. *parvum* and, to a lesser extent, *C*. *hominis* [13]. Dogs and cats are considered reservoirs for human *Cryptosporidium* infections due to their close contact with humans [14].

Cryptosporidiosis is still regarded as a neglected infectious disease in Jordan, with only nine studies conducted in humans and animals [15]. Most of those studies were based on microscopic diagnosis which reported infection rates ranging between 4% and 11% [16, 17]. Drinking water was identified as a source of infection in Jordan on one occasion, and the presence of animals in the area increased the risk of contamination [18].

Hijjawi *et al*. (2010) were the first to conduct a genotyping study in Jordan revealing several rare and novel subtypes in humans [19]. This raised new questions about *Cryptosporidium* transmission in the country. Subsequent molecular typing during a human cryptosporidiosis outbreak demonstrated a high prevalence of *C*. *parvum*, specifically the IIaA17G2R1 subtype [20]. The current study represents the initial report on *Cryptosporidium* infection rates and genotypes/subtypes in dogs in Jordan.

## Materials and methods

### Ethical approval

No invasive methods were employed, and all applicable guidelines for the care and use of animals were followed. The study was approved by the Animal Care and Use Committee (ACUC) at Jordan University of Science and Technology (22-4-2021, Number: 184/2021).

### Sample collection

The sampled dogs were distributed across three governorates (Amman and Zarqa in Central Jordan (COJ) and Irbid in Northern Jordan (NOJ)). The prevalence of *Cryptosporidium* in dogs has not previously been reported in Jordan. Prevalence rates vary considerably, e.g., between 1 and 25%, depending on the study population, region, and detection method [21–25]. Based on the unknown size of the dog population and assuming a prevalence of 20% for *Cryptosporidium*, the targeted sample size was 246 with a precision of 5% and a 95% confidence interval calculated according to Toft and Nielsen (2004) [26].

Between September 2020 and December 2021, a total of 249 dog faecal samples were collected (via cluster random sampling) either by the owner or veterinarian from in-house pet dogs, animals visiting veterinary clinics for checkups, dog shelters, and dog breeding farms. Pet owners provided signed consent for participation.

The samples included 108 strays, 82 pets, and 59 breeding dogs from kennels, ranging in age from one month to nine years (mean and median age, respectively: 22 and 24 months). The questionnaire collected basic information on age, sex, breed, food type, water source, living conditions, and the presence of diarrhoea. Samples were individually sealed and stored at 4°C before being transferred to the Veterinary Parasitology Research Laboratory at Jordan University of Science and Technology. For long-term preservation, the samples were frozen at -20°C until DNA extraction.

### PCR amplification of *Cryptosporidium* DNA

Genomic DNA was extracted from all samples using the QIAamp DNA Soil Mini Kit (Qiagen, Hilden, Germany) following the manufacturer’s protocol, which included mechanical degradation using beads as recommended [27, 28]. DNA was eluted with 50 μl of buffer and stored at -20°C.

A nested PCR assay targeting the *18S rRNA* gene was used to detect *Cryptosporidium* species [29]. A master mix (2x myPOLS, Biotec, Germany) and primary primers were used to amplify a 1325 bp fragment. Subsequently, secondary primers, combined with the same master mix and 2.5 μl of a 1:50 diluted primary PCR product, were used to amplify a fragment ranging from 819 to 825 bp, employing a 58°C annealing temperature for both reactions. In each PCR run, nuclease-free water was used as a negative control, and a *C*. *parvum*-positive cattle sample was confirmed by sequencing and used as a positive control. The visualisation of PCR products was accomplished using a 1.5% agarose gel stained with ethidium bromide.

### *Cryptosporidium* subtyping

Both *C*. *parvum* and *C*. *canis* were genotyped by targeting the *gp60* gene. For *C*. *parvum*, a nested PCR protocol was developed using Solis BioDyne Hot Start master mix (Tartu, Estonia). The primary PCR used the AL3531 and AL3535 primers at a 60°C annealing temperature, which resulted in a 909 bp product. Subsequently, in the secondary PCR, the primary amplicon, combined with the AL3532 and R-LX0029 primers, amplified a 364 bp fragment [30] at a 55°C annealing temperature.

For *C*. *canis*, *gp60* was detected using Promega master mix (Wisconsin, US). GP60-Canis-F1 and GP60-Canis-R1 served as external primers in the nested PCR, while GP60-Canis-F2 and GP60-Canis-R2 acted as inner primers [31]. The annealing temperatures were set at 58°C and 54°C for the primary and secondary PCRs, respectively, resulting in the amplification of 750 bp and 700 bp amplicons.

The *Cryptosporidium gp60* products were excised from the gel and extracted using the Monarch DNA Gel Extraction Kit (BioLabs, New England) following the manufacturer’s instructions.

### Sequencing

The PCR products from the second reaction for the *18S rRNA* and *gp60* genes were subjected to commercial Sanger sequencing (Macrogen, Seoul, South Korea) using a second set of primers for each gene. In addition, a PRA-67DQ *C*. *parvum* strain (ATCC, Virginia, USA) was used to confirm the PCR and sequencing results. The obtained sequences were manually edited and verified using the NCBI Basic Local Alignment Search Tool and aligned with previously reported references available in the GenBank database via ClustalW from BioEdit 7.2.5. Phylogenetic trees (neighbor-joining (NJ), maximum likelihood (ML), and maximum parsimony (MP)) were generated using the Kimura 2-parameter model, and evolutionary analyses were conducted in MEGA11 [32].

### Genetic diversity and differentiation

The genetic structure and differentiation of *Cryptosporidium* populations were evaluated using ARLEQUIN v. 3.5.2.2 and DnaSP v. 5.10 [33]. This evaluation estimated the number of haplotypes (N_H_), private haplotypes, haplotype diversity (H_D_), shared haplotypes (S_H_), nucleotide diversity (π_**D**_), average number of nucleotide differences (k), molecular analysis of variation (AMOVA), fixation index (*F*_ST_) value [34] and Tajima’s D and Fu’s Fs for total individuals. Significance was determined through 1000 permutations. TCS haplotype network maps and median-joining network trees [35, 36] were constructed using the TCS algorithm implemented in PopArt software [37].

### Statistical analysis and generalized linear mixed model

To estimate potential risk factors contributing to *Cryptosporidium* infection, associations between individual risk factors and the outcome were evaluated using the chi-square test in SPSS version 25.0. Odds ratios (ORs) and their corresponding 95% confidence intervals (CIs) were computed with statistical significance and determined at p < 0.05. The conservative Bonferroni correction was used to correct for multiple comparisons.

A generalized linear mixed model (GLMM) was used to evaluate the variables [38], and it incorporates nonnormally distributed variables, nonlinear relationships, and data with dependencies. This model is used to analyze data that involve multiple variables, some of which may be random effects or independent variables of interest (risk factors), and their influence on a response variable (random component), which is the number of *Cryptosporidium* cases detected by *18S rRNA* PCR [39, 40]. The independent variables considered in our investigation included dog type, the location from which the samples were taken, food type, age, sex, presence of diarrhoea, indoor/outdoor, and presence of other animals around or living with the dog.

### GenBank submitted sequences

The nucleotide sequences of the *18S rRNA* gene of *Cryptosporidium* isolated in this study have been deposited in the GenBank database under the following accession numbers: *C*. *parvum*; OP712659.1 to OP712664.1, *C*. *canis*; OQ194022.1 and OQ194023.1, *C*. *baileyi*; OR548275.1, *C*. *parvum gp60*; PP067169 to PP067179, *C*. *canis gp60*; PP067168, and PP083947 to PP083950.

## Results

### Molecular *Cryptosporidium* screening

All 249 faecal samples were screened using *18S rRNA* nested PCR, revealing an overall infection rate of 18.9% (Table 1). According to the chi-square test, no significant differences in infection rates were observed between dog types or collection areas (p>0.05) (Table 1).

**Table 1:** *Cryptosporidium* spp. overall infection rates by region in different dog types tested with *18S rRNA* nested PCR. The mean ($\bar{x}$), standard deviation (SD) and 95% confidence interval (CI) of each factor were calculated.

Dog Type	Amman	Zarqa	Irbid	Total	$\bar{x}$ & SD & CI
	Av. Age = 18 months	Av. Age = 24 months	Av. Age = 27.89 months		Dog Type
**Stray**	11.4% (5/44)	25.0% (14/56)	50.0% (4/8)	21.3% (23/108)	(0.21), (0.411), C. I (0.13–0.29)
**Pet**	20.0% (10/50)	0.0%	18.8% (6/32)	19.5% (16/82)	(0.20), (0.399), C.I (0.11–0.28)
**Breeding**	12.9% (4/31)	0.0%	14.2% (4/28)	13.6% (8/59)	(0.14), (0.345), C.I (0.05–0.23)
**Total**	15.2% (19/125)	25.0% (14/56)	20.6% (14/68)	18.9% (47/249)	
$\bar{x}$ **& SD & CI Region**	(0.15), (0.36), C.I (0.09–0.22)	(0.25), (0.437), C.I (0.13–0.37)	(0.21), (0.407), C.I (0.11–0.30)		

In addition, neither of the risk factors had any significant effect on the *Cryptosporidium* spp. infection rate according to the chi-square 95% Cl test (Table 2).

**Table 2:** PCR test results for *Cryptosporidium* spp., detailing variable types, investigation levels, total tested (n), number of positives, percentages, and p values detected by chi-square test. The mean ($\bar{x}$), standard deviation (SD) and 95% confidence interval (CI) of each factor were calculated.

Variable	level	(n)	Positive	p value	$\bar{x}$ & SD & CI
**Age**	Juvenile (≤ 1 year)	78	14 (17.9%)	0.755*	(0.18), (0.386), C.I (0.09–0.27)
	Young adult (1 < Age ≤ 3)	148	30 (20.3%)		(0.20), (0.403), C.I (0.14–0.27)
	Mature adult (3 < Age ≤ 9)	23	3 (13.0%)		(0.13), (0.344), C.I (-0.02–0.28)
**Gender**	Female	142	27 (19.0%)	0.856	(0.19), (0.394), C.I (0.12–0.26)
	Male	86	17 (19.8%)		(0.20), (0.401), C.I (0.11–0.28)
	Unknown	21	3 (14.3%)		
**In/Out**	Indoor	97	14 (14.4%)	0.152	(0.14), (0.353), C.I (0.07–0.22)
	Outdoor	152	33 (21.7%)		(0.22), (0.1414), C.I (0.15–0.28)
**Food Type**	Raw food	183	36 (19.7%)	0.448	(0.20), (0.399), C.I (0.14–0.25)
	Cooked or processed	59	9 (15.3%)		(0.15), (0.363), C.I (0.06–0.25)
	Unknown	7	2 (28.6%)		
**Diarrhoea**	Yes	65	8 (12.3%)	0.115	(0.12), (0.331), C.I (0.04–0.21)
	No	184	39 (21.2%)		(0.21), (0.410), C.I (0.15–0.27)
**Other animals**	Yes	189	35 (18.5%)	0.463	(0.19), (0.389), C.I (0.13–0.24)
	No	52	12 (23.1%)		(0.23), (0.425), C.I (0.11–0.35)
	Unknown	8	0		

*One or more cells have less than 5. Fisher Exact test

### *18S rRNA* sequencing and species identification

**Fig 1:**
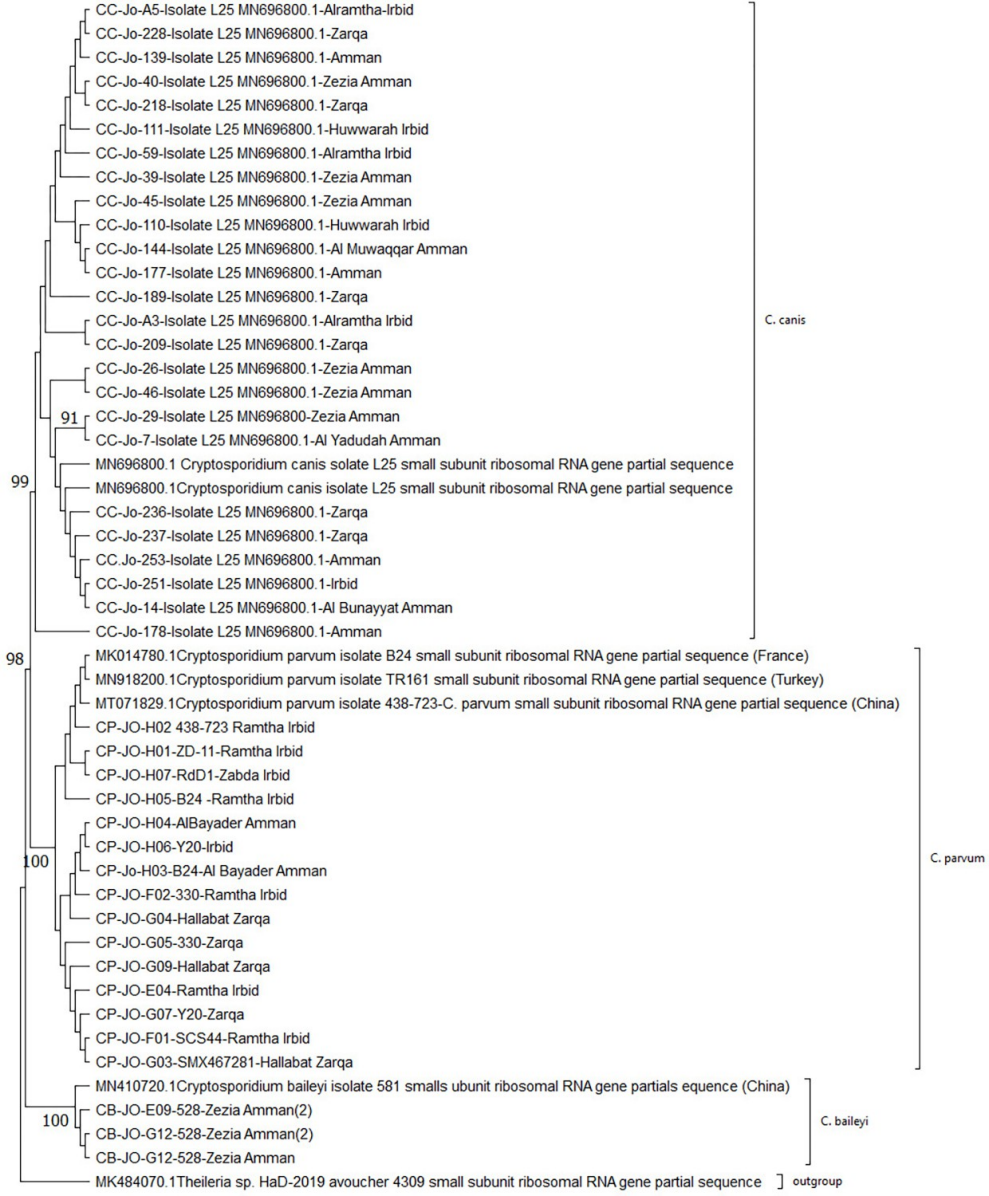
Maximum-likelihood phylogenetic tree of *Cryptosporidium* spp. using *18S rRNA* sequencing results, analysed with the Kimura 2-parameter model and evolutionary analyses in MEGA11.

Among the *18S rRNA* PCR products, successful sequencing was achieved for 43 samples: 15 identified as *C*. *parvum*, 25 as *C*. *canis*, and 3 as *C*. *baileyi* (Table 3 and Fig 1). Phylogenetic analysis was performed for all *18S rRNA* sequences, and an outgroup of genetically related parasites (*Theileria_sp*., MK484070.1) was used to root the tree. The ML phylogenetic tree was constructed using the 43 sequences based on the 730 bp of the nuclear *18S rRNA* sequences (Fig 1).

**Table 3:** Distribution of *Cryptosporidium* spp. sequenced by the *18S rRNA* gene *gp60*. The *Cryptosporidium* species, number of isolates, accession number of deposited sequences (*18S rRNA*), host, identity matches were determined using BLAST, and the deposited sequence accession numbers were assigned according to the *gp60* gene.

Sample #	*Species*	Best match Acc. #	Identity	18s rRNA	#	gp60	Subtype
F1	*C*. *parvum*	MF327254.1	99.47%	OP712661	1	ND	
F2	*C*. *parvum*	MK121775.1	99.74%	ND	1	PP067172	IId
G3	*C*. *parvum*	MN379944.1	99.47%	ND	1	PP067176	IId
G4	*C*. *parvum*	MK301253.1	99.74%	OP712660	1	PP067170	IId
G5	*C*. *parvum*	MK121775.1	99.74%	ND	1	PP067177	IId
G7	*C*. *parvum*	KJ917579.1	99.34%	OP712663	1	PP067178	IId
G9	*C*. *parvum*	MK301253.1	99.47%	ND	1	PP067175	IId
G10	*C*. *parvum*	MK301253.1	98.41%	ND	1	PP067179	IId
H1	*C*. *parvum*	KF128755.1	99.48%	OP712664	1	PP067169	IId
E4	*C*. *parvum*	MT071829.1	99.60%	OP712662	1	PP067174	IId
H4	*C*. *parvum*	MK301253.1	99.47%	OP712659	1	PP067171	IId
H6	*C*. *parvum*	KJ917579.1	99.48%	ND	1	PP067173	IId
H5, H3	*C*. *parvum*	MK014780.1	99.34%	ND	2	ND	
H7	*C*. *parvum*	KP004204.1	99.74%	ND	1	ND	
A5	*C*. *canis*	MN696800.1	99.60%	ND	1	PP083947	XXd1
111	*C*. *canis*	MN696800.1	99.87%	OQ194022	20	ND	
139	*C*. *canis*	MN696800.1	99.87%	ND	1	PP083948	XXe2
178	*C*. *canis*	MN696800.1	99.47%	ND	1	PP083949	XXe1
218	*C*. *canis*	MN696800.1	99.60%	ND	1	PP083950	XXe2
236	*C*. *canis*	MN696800.1	100.00%	ND	1	PP067168	XXe2
251	*C*. *canis*	MN696800.1	99.87%	OQ194023	1	ND	
G12	*C*. *baileyi*	MN410723.1	99.74%	OR548275.1	3	NT	

ND: Not deposited, NT: not tested

After *18S rRNA* sequencing and species identification, risk factors were studied. According to the chi-square test, no significant associations were detected between risk factors and the species identified, except for living with other animals (p = 0.043) (S1 Table). Among the dogs infected with *C*. *parvum*, one lived with cattle, while five others cohabited with cats, three of which were also with horses, deer, and ostriches. Additionally, dogs infected with *C*. *baileyi* were found to have consumed raw food, two indoors and one outdoors.

### Variability of DNA sequences

Nuclear *18S rRNA* sequences obtained from 15 *C*. *parvum* and 25 *C*. *canis* isolates were aligned. The sequences of the isolated strains and reference strains from GenBank contained a 733 bp fragment encompassing 613 conserved sites and 127 variable sites, 27 of which were parsimony informative.

The *18S rRNA* gene sequence showed a mean intraspecific divergence ranging from 0% to 0.022%. The highest observed divergence within a single species was 0.031%, where the divergence was <3.1% in most cases (S2 Table). Among the different species, the divergence ranged from 2.9% to 4.83% (S3 Table).

The *18S rRNA*-ML tree revealed a similar topology for NJ and MP and revealed three main clades, each containing different isolates of each species from various locations. Populations from the NOJ and COJ populations were mixed and did not exhibit distinct branches based on geographical distribution.

**Fig 2:**
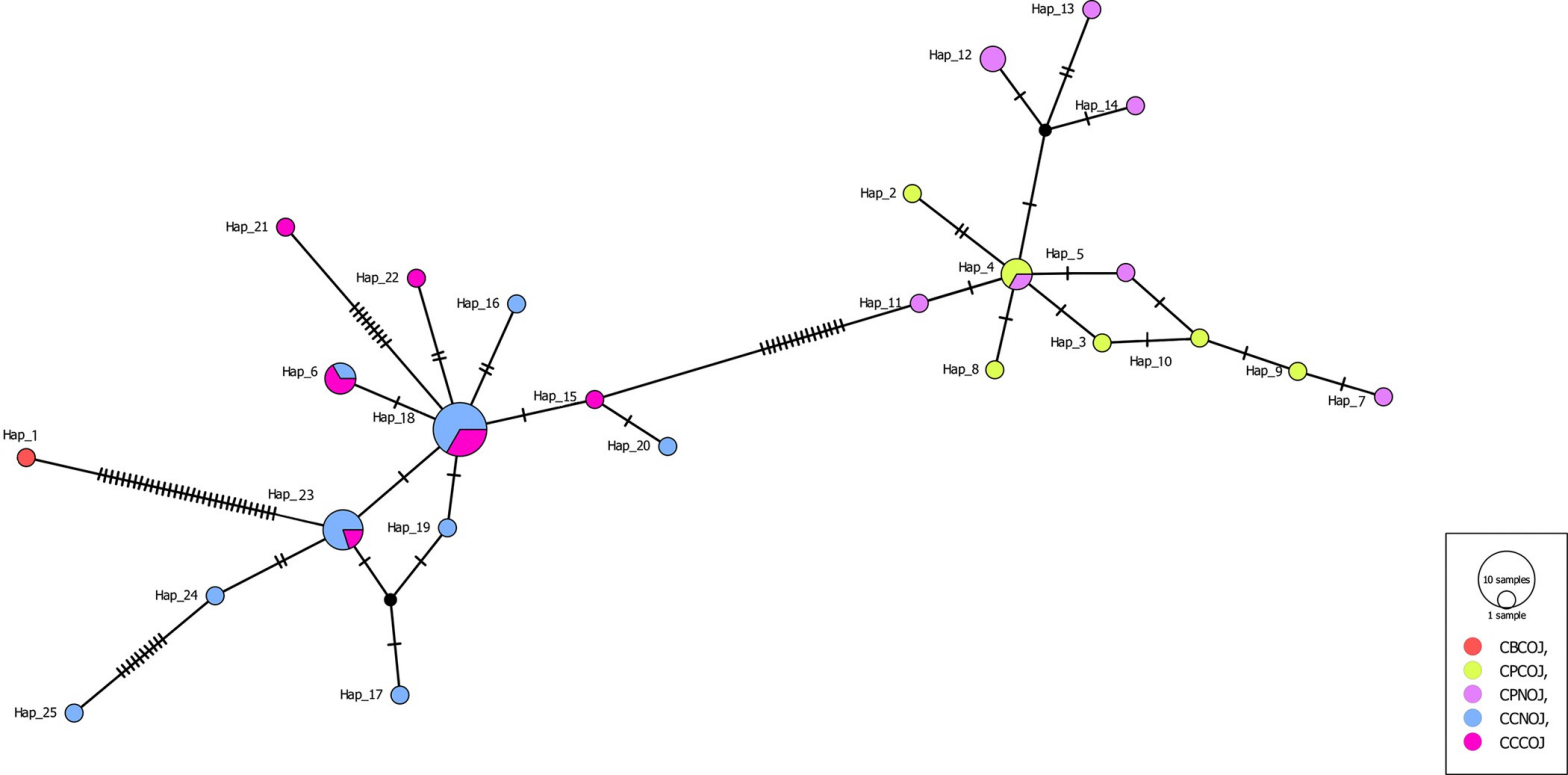
TCS haplotype network of *Cryptosporidium* spp. based on the *18S rRNA* gene from two different populations in Jordan.

The haplotype tree of the *C*. *parvum* and *C*. *canis* populations revealed the presence of a single dominant haplotype surrounded by closely related singletons in a star-shaped configuration. The network based on the *18S rRNA* haplotypes displayed a pattern comparable to that of the phylogenetic tree (Fig 2).

The haplotype and nucleotide diversities for *C*. *canis* in the two populations were 0.813 and 0.002970, respectively. Similarly, for *C*. *parvum* in the two populations, the haplotype and nucleotide diversities were 0.871 and 0.003270, respectively (Table 4).

**Table 4:** Summary of the polymorphisms and neutrality test statistics for the *18S rRNA* gene from *Cryptosporidium* spp.

Species	Region	N	Hd	Pi	Tajima’s D	P value	Fu& Fs	P value	Nm
*C*. *canis*	Total	25	0.813	0.002970					200.05
	COJ	19			-2.0662	0.005	-0.750	0.368	
	NOJ	6			-1.2950	0.069	-0.1683	0.342	
*C*. *parvum*	Total	15	0.871	0.003270					40.96
	COJ	7			0.23902	0.600	-1.784	0.08	
	NOJ	8			-0.4511	0.330	-1.789	0.10	

n: number of sequences, Hd: haplotype diversity, Pi: Nucleotide diversity, Tajima’s D and Fu’s Fs: Neutrality tests, Nm: gene flow

### *C*. *parvum* genetic differentiation and structure

In this study, *C*. *parvum* exhibited low nucleotide diversity (0.0032) and high haplotype diversity (0.871), indicating a high number of closely related haplotypes. Tajima’s D test estimated neutrality at -0.451 for the NOJ and 0.239 for the COJ. The nonsignificant negative values of the NOJ population suggest an extra infrequent nucleotide variant compared to what would be expected under a neutral model, indicating genetic equilibrium. Moreover, the positive nonsignificant Tajima’s D test in the COJ population suggested an excess of intermediate frequency alleles consistent with balancing selection or population decline. The Fuʼs Fs test values (Table 4) were negative and nonsignificant for the NOJ and COJ populations (-1.789 and -1.784, respectively), revealing the presence of infrequent haplotypes beyond what would be expected under a neutral model.

Additionally, a low *F*_ST_ value (0.01206) was found between the two populations (Table 5), indicating low genetic differentiation. The Nm values among the two populations were greater than 1 (40.96) (Table 5), suggesting frequent gene exchange or probable high gene flow, causing low genetic differentiation within populations [41].

**Table 5:** AMOVA of the *18S rRNA* gene of the *Cryptosporidium* spp. population.

Species	Source of variation	Degrees of freedom	Sum of squares	Variance components	Percentage of variation	Fixation Index (*F*_ST_)
*C*. *canis*	Among population	1	1.676	0.00249 Va	0.249	0.00249
	Within population	23	37.684	1.6384 Vb	99.750	
	Total	24	39.360	1.642		
*C*. *parvum*	Among population	1	5.787	0.064 Va	1.21	0.01206
	Within population	13	68.945	5.303 Vb	98.79	
	Total	14	74.733	5.368		

Typically, the haplotype at the centre of the network is considered the oldest, while the more recent haplotypes are situated at the network’s edges. In this study, both the *C*. *canis* and *C*. *parvum* (Fig 2) haplotypes are depicted as star-shaped networks, with Hap 4 located at the centre for *C*. *parvum* and Hap 18 being the most abundant for *C*. *canis*; these haplotypes are also positioned at the network’s centre. In *C*. *canis*, other haplotypes had mutations from Hap 18 (appearing five times) into eight other haplotypes and one missing haplotype, consistent with the significant neutrality test signifying recent population expansion in the COJ population, represented by four specimens. The star-like network suggested few mutations between haplotypes. Most individuals possess unique haplotypes, with the majority found in only one location (private haplotypes), and only three haplotypes are shared among sites. This suggests substantial subdivision among individuals within populations, strong recent population expansion or both, aligning with other statistical indices in this study. The dominant central haplotype shared isolates from the two geographic populations, indicating genetic stability and likely adaptation to the environment compared to the remaining haplotypes.

Finally, molecular variance analysis (AMOVA) demonstrated that most of the genetic variance occurred within populations (98.79%) (Table 5), while only 1.2% appeared among populations.

### *C*. *canis* genetic differentiation and structure

The neutrality test for *C*. *canis*, estimated using Tajima’s D test (Table 4), yielded negative and nonsignificant values for the NOJ population (-1.295), indicating genetic equilibrium. However, for the COJ population, a significantly negative value was shown (-2.066), suggesting an excess of rare nucleotide variants. The Nm value of 200.05, according to Low *et al*. (2014), indicates high gene flow between populations [42]. High gene flow implies low genetic differentiation, reflecting the homogeneity of the two populations and low potential for migration. Furthermore, Fu’s Fs values were not significantly negative, indicating that the population had not recently experienced expansion. The obtained pairwise ***F***_**ST**_ value between the two populations was 0.00249, indicating low genetic differentiation (Table 4). AMOVA revealed that most of the genetic variance occurred within populations (99.75%), while only 0.25% of the variance occurred between the two populations.

### GLMM outcomes

The run test made on the data excluding stray dogs revealed that (age group 1<AGE≤3, raw food consumption, pet dogs, living with other animals, female, indoor, and Amman dogs) variables were acceptable for analysis via the GLMM test. According to the GLMM, raw food consumption, the presence of other animals nearby, and indoor/outdoor dogs variables were significantly associated with cryptosporidiosis in dogs.

The run test was performed on all the data, including stray dog data, and revealed that the type of dog, indoor/outdoor, food type, age, and living with other animals could be analysed via the GLMM test. Considering indoor/outdoor, food type, and living with other animals are not appropriate for analysis at the whole data level since stray dogs are always outdoors, eating raw food and living or interacting with different animals. Dog type and age were not significantly associated with the infection rate according to GLMM.

The model highlights the importance of demographic and environmental factors. These findings could be useful for further studies on the impact of these variables on *Cryptosporidium* infection, potentially guiding public health policies or clinical recommendations. Negative coefficients, such as being in the younger age group (1<Age>3), absence of pets (breeding dogs), being male, and being outdoors, are associated with a decrease in *Cryptosporidium* infection levels. Positive coefficients representing variables such as consuming raw food, presence of other animals, and living in Amman were associated with an increase in *Cryptosporidium* infection levels (Table 6). The final outcome could be summarized as follows: the infection rate was significantly associated with dogs living with other animals, raw food consumption and indoor dog status (Table 6).

**Table 6:** Generalized linear mixed model (GLMM) of dog risk factor variables associated with *Cryptosporidium* spp. infection rate using *18S rRNA* nested PCR.

Fixed Coefficients									
Model Term	Coeff	Std. Error	t	Sig.	95% Confidence Interval		Exp	95% Confidence Interval for Exp	
					Lower	Upper		Lower	Upper
**Intercept**	2.10	.71	2.95	.005	.658	3.54	8.17	1.93	34.59
**1<Age>3 = 0**	-.48	.55	-.87	.390	1.60	.64	.62	.202	1.89
**1<Age>3 = 1**	0^b^	.	.	.	.	.	.	.	.
**RawFood = 0**	1.56	.74	2.11	.041	.064	3.05	4.75	1.07	21.15
**RawFood = 1**	0^b^	.	.	.	.	.	.	.	.
**Pet = 0**	.56	.60	.94	.355	-.65	1.78	1.75	.52	5.91
**Pet = 1**	0^b^	.	.	.	.	.	.	.	.
**OtherAnimals = 0**	1.42	.64	2.20	.034	2.72	.115	.24	.066	.89
**OtherAnimals = 1**	0^b^	.	.	.	.	.	.	.	.
**Indoor = 0**	-1.25	.604	2.07	.045	2.47	.027	.29	.084	.97
**Indoor = 1**	0^b^	.	.	.	.	.	.	.	.
**Female = 0**	.046	.528	.087	.931	1.02	1.12	1.05	.359	3.05
**Female = 1**	0^b^	.	.	.	.	.	.	.	.
**Amman = 0**	.20	.656	.305	.762	1.13	1.53	1.22	.324	4.60
**Amman = 1**	0^b^	.	.	.	.	.	.	.	.

Probability distribution: Binomial

Link function: Logit^a^

a. Target: Cryp18S

b. This coefficient is set to zero because it is redundant.

### *Cryptosporidium gp60* genotyping

Samples identified through *18S rRNA* sequencing as *C*. *parvum* and *C*. *canis* were subjected to further investigation by amplifying the *gp60* gene and sequencing the amplified fragments to accurately identify the subtypes present in Jordan. Among the *C*. *parvum* samples, 14 were successfully sequenced and identified as IId subtypes (Table 3). Four subtypes were detected, namely, IIdA18G1, IIdA19G1, IIdA20G1, and IIdA22G1, with IIdA20G1 being the dominant subtype (63.36%). Additionally, five *C*. *canis* samples were successfully sequenced, revealing the XXd1 (one sample), XXe1 (one sample) and XXe2 (three samples) subtypes (Table 3).

## Discussion

The present study represents the first molecular screening for *Cryptosporidium* spp. infecting dogs in Jordan. Three species were reported, one common zoonotic species (*C*. *parvum* subtype IId), one bird-specific species (*C*. *baileyi*), marking the first report of its occurrence in dogs globally, and lastly, one dog-specific species (*C*. *canis* subtypes XXe and XXd).

Globally, the average *Cryptosporidium* infection rate in dogs has been reported to be 6% (95% CI: 4–9%) according to nested *18S rRNA* PCR [5]. In this study, the overall infection rate (18.9%) of *Cryptosporidium* spp. in dogs in the selected regions of Jordan was relatively high. This finding is comparable to the prevalence in Japan (21% in kennel dogs) [43] and Egypt (24%) [44], but lower than the rates reported in Iraq (28.6% and 47%) [45, 46]. However, these rates were higher than the infection rates reported in Canada (6% and 2.4%) [47, 48], the USA (7%) [49], China (ranging from 1.6% to 8%) [50, 51], Italy (2.5%) [52], Brazil (7.8%) [53], and Thailand (ranging from 0.7% to 12.1%) [54, 55].

The variations in prevalence among countries and different studies can be explained not only by factors such as dog type, age, origin, health status, and examination methods used [56] but also by environmental factors in the regions that sustain the parasite, making them accessible for animal infections. The true prevalence might also vary due to the variability in shedding from animals over time and in quantity, in addition to the sensitivity of PCR amplification [57].

In this study, the infection rate of *Cryptosporidium* (detected by *18S rRNA* nested PCR) was significantly associated with the presence of *Cryptosporidium* in indoor dogs, most of which were pet or breeding dogs. Previous studies have shown that pet dogs have a greater risk of *Cryptosporidium* spp. infection [58]. Additionally, pets in general are more susceptible to infectious diseases [59]. Conversely, it was significantly associated with dogs cohabiting with other animals, encompassing breeding and some pet dogs. Although raw food consumption seems to be an associated risk factor, a negative relationship was found in this study, which is similar to the findings of other studies. *Cryptosporidium* species are more closely associated with water sources because they are transmitted through water [60].

Since there was no significant association between *Cryptosporidium* spp. infection and diarrhoea in dogs analyzed, these findings reinforce the idea that cryptosporidiosis in companion animals is characteristically asymptomatic [61], which results in dogs that do not show clinical signs and therefore are not treated have a greater zoonotic and epidemiological importance and pose greater public health risks, especially to immunocompromised humans and animals [62].

Sequencing the secondary PCR products from the *Cryptosporidium*-positive samples revealed that 25 *C*. *canis* (58.1%), 15 *C*. *parvum* (34.9%), and three *C*. *baileyi* (7.0%). *C*. *canis*, a dog-specific species, can be found in both pets and their owners [63]. This specie is reported relatively frequently in humans [12], including people from China [64] and Jordan [19]. Recently, *C*. *canis* was the primary parasite found (94.3% of *Cryptosporidium*-positive samples) in young dogs in Germany [65] and in farmed minks, raccoon dogs, and foxes in China [3].

In our study, three dogs were infected with C. *baileyi* (a bird-specific species). Two were pets from Amman, living indoors and consuming uncooked food, they had diarrhoea without other animals around. The third dog, a stray from Zarqa, lived outdoors and consumed uncooked food. While *C*. *baileyi* has been previously reported in chicken samples from Jordan [66], infection in dogs has never been reported anywhere, and it is rarely transmitted from birds to other hosts. Its presence in dogs in Jordan might be attributed to the consumption of raw chicken meat.

*C*. *parvum* was found at a higher infection rate in dogs living with other animals (p-value = 0.043). Subsequently, infected dogs may transmit the infection to other animals and people. In industrialised countries, human cryptosporidiosis often results from contact with infected humans or animals, in addition to international travel.

However, a study in Egypt on humans and dogs sharing households or family farms confirmed a high probability of zoonotic transmission of *C*. *parvum* between children and dogs [44, 67]. Similarly, in Jordan, the presence of zoonotic species and subtypes could be due to several risk factors that are shared between the two countries, including high numbers of stray dogs, pet dogs being kept outdoors, improper disposal of animal waste, and drinking contaminated surface water.

Within the genome of *Cryptosporidium* spp., the *gp60* gene represents the marker with the largest identified polymorphism [68]. *C*. *parvum* has been genotyped into several subtypes, three of which are known to be transmissible to humans; both the IIa and IId subtypes from ruminants are zoonotic, while the IIc subtype is anthroponotic [69]. In this study, all the sequenced *C*. *parvum* isolates belonged to the IId subtype, marking, to the best of our knowledge, the first report of this subtype infecting dogs worldwide. In this study, four subtypes were detected. The identified IIdA20G1 subtype has been previously found in various animals, e.g., *Corvus* [70], and has caused numerous outbreaks in calves, including those in large cattle farms in China [71]. Additionally, it has been reported in livestock and humans worldwide, with numerous reports from the Middle East [72–75]. The IIdA20G1 subtype was previously reported to infect humans in Jordan [19, 66]. Human infections by the other subtypes have been reported in several studies from different parts of the world, including Spain [76] and among hospitalised children in Qatar [77], possibly indicating the zoonotic nature of these subtypes.

Genotyping of *C*. *canis* through the *gp60* gene identified XXd1, XXe1 and XXe2 subtypes, which are considered canine-specific [78]. According to the *Cryptosporidium* spp. *18S rRNA* phylogenetic analysis, the minimum interspecific divergence was low. Generally, the divergence within species for the *18S rRNA* gene is less than the genetic distance observed between species.

In this study, both tested species in two different regions exhibited low nucleotide and high haplotype diversity values, indicating that a high number of haplotypes suggested recent population expansion. These findings are consistent with those of Tajima’s D and Fu’s Fs, which were performed to detect past population growth. The overall negative values of both neutrality tests within the two species and regions indicate an excess of rare alleles and haplotypes, implying recent population expansion. This was confirmed by the TCS haplotype tree, which displayed major haplotypes surrounded by singletons. The nucleotide variation within the NOJ of *C*. *parvum* could be due to locally generated mutations during *C*. *parvum* infection resulting from the sexual phase of the life cycle. This allows recombination between genetically variable strains, leading to the evolution of new subtypes and adaptation [79].

Furthermore, the Nm levels indicate very high gene flow for both *C*. *canis* and *C*. *parvum* [80], suggesting that high gene flow or high genetic exchange between populations may occur and lead to low genetic differentiation between subpopulations. Additionally, high gene flow is supported by low pairwise *F*_ST_ values (Table 5). Based on Wright (1978), genetic differentiation is considered low when the *F*_ST_ is less than 0.05 [81], which was the case in this study. These results coincided with the AMOVA results (Table 5), where most of the variations were within-population. The AMOVA results revealed little variation among the geographical regions. This finding suggested that the geographical expansion of *C*. *canis* and *C*. *parvum* through the movement and migration of dogs may explain the genetic exchanges observed within these populations.

## Conclusions

Dog owners should aim at keeping their dogs in a closed, clean environment. Infected dogs should be isolated to reduce the risk of transmission to sensitive populations, such as puppies and young children [82].

*Cryptosporidium parvum*-positive samples genotyped by *gp60* were identified as the zoonotic genotype IId, comprising four subtypes (IIdA20G1, IIdA22G1, IIdA18G1, and IIdA19G1) previously found in various animals and humans globally. This poses a public health risk, highlighting the potential for dogs to transmit *C*. *parvum* in Jordan. Since dog owner infection status was not tested in the present study, firm conclusions on the risk of *C*. *parvum* transmission to humans cannot be drawn. However, considering the global reports and zoonotic nature of these subtypes, greater precaution is warranted, especially among immunocompromised individuals.

## Supporting information

S1 Table Distribution of *Cryptosporidium* spp. with risk factors. (DOCX)

S2 Table Estimates of evolutionary divergence between sequences. Analyses were conducted using the Kimura 2-parameter model and the MEGA11 program.
(XLSX)

S3 Table Estimation of evolutionary divergence across sequence pairs between populations. Analyses were conducted using the Kimura 2-parameter model and the MEGA11 program.
(XLSX)
