# Supplementary material for: Molecular identification and genetic diversity analysis of Cryptosporidium spp. infecting dogs from central and northern Jordan: Detection of zoonotic genotype IId
Source: PLoS One. 2025 Feb 6;20(2):e0314462. doi: 10.1371/journal.pone.0314462 (PMC11801568; doi:10.1371/journal.pone.0314462)
Supplement: S1 Table — (DOCX) [file pone.0314462.s001.docx]

|  |  | *C. canis* Out of 25 | *C. parvum* Out of 15 | *C. baileyi* Out of 3 | | | P value |  |
| --- | --- | --- | --- | --- | --- | --- | --- | --- |
| Dog Type | Stray | 9 (36.0%) | 10 (66.7%) | 1 (33.33%) | | | 0.155* |  |
|  | Pet | 11 (44.0%) | 2 (13.3%) | | 2 (66.67%) | |  |  |
|  | Breeding | 5 (20.0%) | 3 (20.0%) | | 0 (0%) | |  |  |
| In/out | Indoor | 10 (40.0%) | 2 (13.3%) | | | 2 (66.67%) | 0.093* |  |
|  | Outdoor | 15 (60.0%) | 13 (86.7%) | | | 1 (33.33%) |  |  |
| Food Type | Raw food | 21 (84.0%) | 11 (73.3%) | | | 3 (100%) | 0.289* |  |
|  | Cooked or processed food | 2 (8.0%) | 4 (26.7%) | | | 0 (0%) |  |  |
|  | Unknown | 2 (8.0%) |  |  |  |  |  |  |
| Age | Age ≤1 | 6 (24.00%) | 5 (3.33%) | | | 1 (33.33%) | 0.738* |  |
|  | 1<Age <3 | 16 (64.00%) | 10 (66.66%) | | | 2 (66.60%) |  |  |
|  | Age > 3 | 3 (12.00%) | 0 (00.00%) | | | 0 (00.00%) |  |  |
| Diarrhoea | Yes | 4 (16.00%) | 2 (12.50%) | | | 2 (66.66%) | 0.134* |  |
|  | No | 21 (84.00%) | 13 (86.66%) | | | 1 (33.33%) |  |  |
| Gender | Female | 13 (52.0%) | 13 (86.66%) | | | 3 (100%) | 0.113* |  |
|  | Male | 9 (36.0%) | 2 (13.33%) | | | 0 (0%) |  |  |
| Other animals | Yes | 16 (64.00%) | 14 (93.33%) | | 1 (33.33%) | | 0.043* |  |
|  | No | 9 (36.00%) | 1(6.66%) | | 2 (66.67%) | |  |  |
| *One or more cells have less than 5. Fisher Exact test | | | | | | | | |
